# Prognostic marker for severe acute exacerbation of chronic obstructive pulmonary disease: analysis of diffusing capacity of the lung for carbon monoxide (D_LCO_) and forced expiratory volume in one second (FEV_1_)

**DOI:** 10.1186/s12890-021-01519-1

**Published:** 2021-05-06

**Authors:** Juwhan Choi, Jae Kyeom Sim, Jee Youn Oh, Young Seok Lee, Gyu Young Hur, Sung Yong Lee, Jae Jeong Shim, Chin Kook Rhee, Kyung Hoon Min

**Affiliations:** 1 Division of Pulmonary, Allergy and Critical Care Medicine, Department of Internal Medicine, Korea University Guro Hospital, Korea University College of Medicine, 148, Gurodong-ro, Guro-gu, Seoul, 08308 Republic of Korea; 2 Division of Pulmonary, Allergy and Critical Care Medicine, Department of Internal Medicine, Seoul St. Mary’s Hospital, College of Medicine, The Catholic University of Korea, 222 Banpo-daero, Seocho-gu, Seoul, 06591 Republic of Korea

**Keywords:** COPD, D_LCO_, FEV_1_

## Abstract

**Background:**

It is important to assess the prognosis of patients with chronic obstructive pulmonary disease (COPD) and acute exacerbation of COPD (AECOPD). Recently, it was suggested that diffusing capacity of the lung for carbon monoxide (D_LCO_) should be added to multidimensional tools for assessing COPD. This study aimed to compare the D_LCO_ and forced expiratory volume in one second (FEV_1_) to identify better prognostic factors for admitted patients with AECOPD.

**Methods:**

We retrospectively analyzed 342 patients with AECOPD receiving inpatient treatment. We classified 342 severe AECOPD patients by severity of D_LCO_ and FEV_1_ (≤ vs. > 50% predicted). We tested the association of FEV_1_ and D_LCO_ with the following outcomes: in-hospital mortality, need for mechanical ventilation, need for intensive care unit (ICU) care. We analyzed the prognostic factors by multivariate analysis using logistic regression. In addition, we conducted a correlation analysis and receiver operating characteristic (ROC) curve analysis.

**Results:**

In multivariate analyses, D_LCO_ was associated with mortality (odds ratio = 4.408; 95% CI 1.070–18.167; *P* = 0.040) and need for mechanical ventilation (odds ratio = 2.855; 95% CI 1.216–6.704; *P* = 0.016) and ICU care (odds ratios = 2.685; 95% CI 1.290–5.590; *P* = 0.008). However, there was no statistically significant difference in mortality rate when using FEV_1_ classification (*P* = 0.075). In multivariate linear regression analyses, D_LCO_ (*B* = − 0.542 ± 0.121, *P* < 0.001) and FEV_1_ (*B* = − 0.106 ± 0.106, *P* = 0.006) were negatively associated with length of hospital stay. In addition, D_LCO_ showed better predictive ability than FEV_1_ in ROC curve analysis. The area under the curve (AUC) of D_LCO_ was greater than 0.68 for all prognostic factors, and in contrast, the AUC of FEV_1_ was less than 0.68.

**Conclusion:**

D_LCO_ was likely to be as good as or better prognostic marker than FEV_1_ in severe AECOPD.

## Background

Chronic obstructive pulmonary disease (COPD) is a chronic airway disease defined by persistent respiratory symptoms and irreversible airflow limitation [1–3]. Patients with COPD present with various symptoms, such as cough, sputum, and dyspnea, and these symptoms are closely related to the quality of life and prognosis [4, 5]. The global initiatives for chronic obstructive lung disease (GOLD) reports emphasize treatment based on patient history and symptoms, such as exacerbation history, the modified medical research council dyspnea scale (mMRC), and COPD assessment test (CAT) [6]. Forced expiratory volume in one second (FEV_1_) is still used to grade the severity of airflow obstruction, but the 'refined ABCD assessment tool' excludes FEV_1_ from the criteria for evaluating the 'ABCD' group. This is because the FEV_1_ value is weakly correlated with the patient's symptoms and health status [7, 8]. However, pulmonary function tests (PFT) are still important tests for diagnosing and treating COPD in the clinical field. Therefore, we want other PFT factors related to the patient's symptoms and health status rather than FEV_1_. Several studies have shown that the diffusing capacity of the lung for carbon monoxide (D_LCO_) among the various values of PFT is closely related to patient symptoms, prognosis, and oxygen demand in COPD [9, 10]. In addition, there was a recent opinion that D_LCO_ should be added to multidimensional tools assessing COPD [11]. This study aimed to compare FEV_1_ and D_LCO_ through the prognosis of severe acute exacerbations of COPD (AECOPD).

### Method

## Study population

We retrospectively analyzed the medical records of 342 patients admitted to Korea University Guro Hospital from January 2011 to May 2017. We searched our electronic medical records database with the keywords “COPD” and “Acute exacerbation.” This study was approved by the Institutional Review Board of Korea University Guro Hospital (KUGH16131-002). The requirement for informed consent from the patients was waived due to the retrospective nature of this study by the institutional review committee.

All patients included only patients who were followed up for more than 1 year in our hospital under the diagnosis of COPD. COPD and airflow limitation were diagnosed by synthesizing patient-reported respiratory symptoms, PFT (the ratio of FEV_1_ to forced vital capacity (FVC) was less than 70% in post-bronchodilator spirometry), chest image, and patient`s history (smokers with at least ten pack-years of tobacco exposure, etc.) by an experienced pulmonologist [6]. AECOPD was defined as worsening of the patient’s respiratory symptoms beyond normal day-to-day variation. Severe AECOPD was defined as ‘if the patient needs hospitalization due to AECOPD.’ The spirometry data used in the analysis was previously performed in the outpatient clinic during the stable period. Spirometry value that was measured within 1 year from the hospitalization day were used. Patients were excluded with the following criteria: (1) the cause of admission was not AECOPD; for example, acute heart failure, acute pulmonary edema, acute pulmonary embolism, pneumothorax, and arrhythmia (These diseases were excluded through cardiac enzyme, electrocardiogram, echocardiogram and chest image.), (2) the patient had undergoing active cancer treatment, (3) the patient received a major operation within 3 months, (4) the patient had an acute coronary syndrome, brain hemorrhage, or brain infarction within 3 months, (5) the patient had previously been diagnosed with asthma, and (6) the patient had no D_LCO_ results. All patients were 40 years old or older. We retrospectively analyzed the charts by two experienced pulmonologists to exclude various exclusion factors. "events" is synonymous with "patients" in this study.

**Fig. 1:**
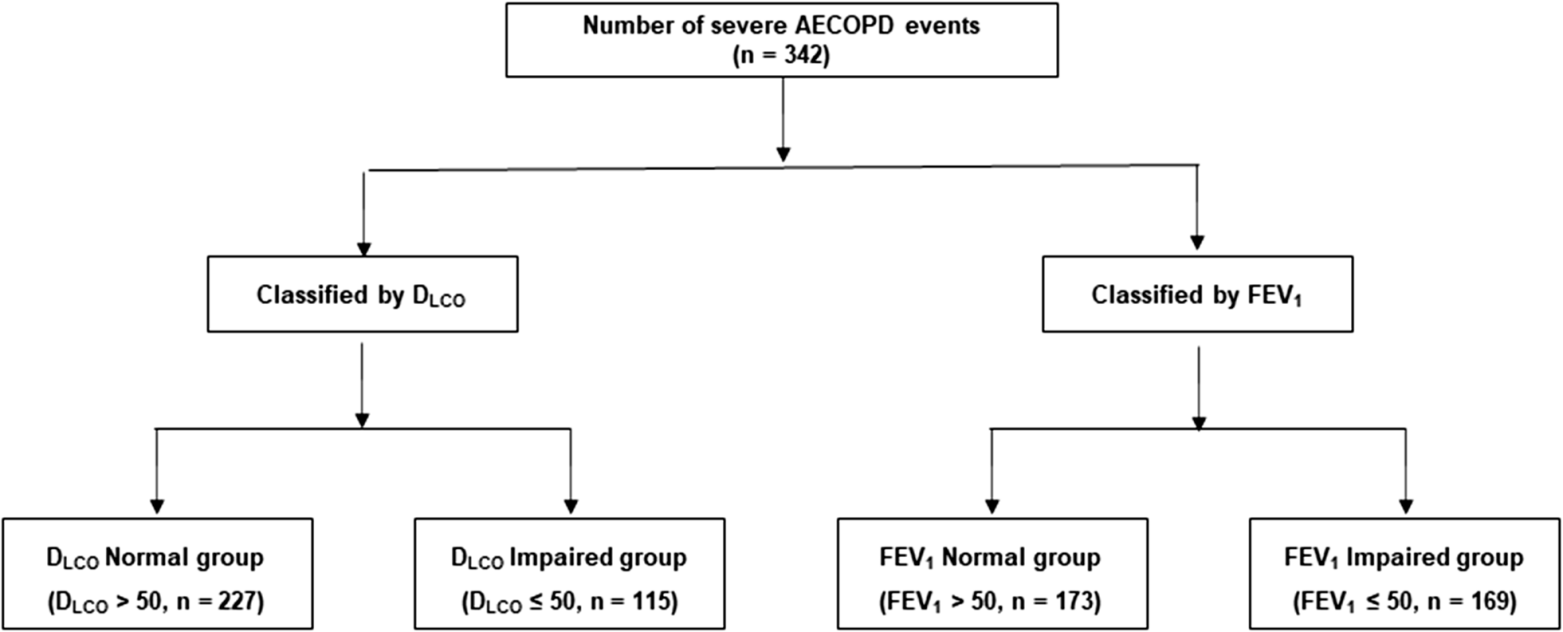
Study design

We classified 342 severe AECOPD patients by severity of D_LCO_ and FEV_1_ (≤ vs. > 50% predicted). When the D_LCO_ value is more than 50 (% of predicted value), it is defined as the 'D_LCO_ normal group' and when it is 50 (% of predicted value) or less, it is defined as the 'D_LCO_ impaired group' [11]. Likewise, when the FEV_1_ value is more than 50 (% of predicted value), it is defined as the ‘FEV_1_ normal group' and when it is 50 (% of predicted value) or less, it is defined as the ‘FEV_1_ impaired group' (Fig. 1).

### Data collection

We tested the association of FEV_1_ and D_LCO_ with the following outcomes: in-hospital mortality, need for mechanical ventilation, need for intensive care unit (ICU) care. When the patient was hospitalized more than once, only the first hospitalized events were included, and the others were excluded. The following medical data were analyzed: age, sex, smoking history, comorbidities, baseline spirometry, inhaler and oral medication before admission, length of hospital stay, hospital mortality, experience of mechanical ventilation, and experience of ICU care in hospital.

### Statistical analysis

Data were analyzed using SPSS 20 software (SPSS for Windows, SPSS Inc., Chicago, IL, USA). Data are presented as average ± standard deviation or number (percentage). We performed a statistical analysis in two directions. First, two groups were classified using D_LCO_ and FEV_1_ and analyzed statistically. Continuous variables were compared using the independent t-test, and categorical variables were compared using the chi-squared test. We analyzed the prognostic factors (except length of hospital stay) by multivariate analysis through logistic regression. Multivariate analysis was conducted for variables with a *P *value of less than 0.05 in the univariate analysis, except for baseline spirometry (D_LCO_ and FEV_1_). In the case of D_LCO_, multivariate analysis included sex, previous TB history, cerebrovascular accident, inhaler use before admission, oral β2 adrenoreceptor agonist, roflumilast, and mucolytic agent. In the case of FEV_1_, multivariate analysis included age, sex, previous TB history, inhaler use before admission, roflumilast, and mucolytic agent. Multivariate analysis was conducted using a backward elimination procedure and was assessed by the Hosmer–Lemeshow test.

Second, the linear correlation between spirometry factors (D_LCO_ and FEV_1_) and length of hospital stay were analyzed. In univariate analysis, the correlation coefficients between spirometry factors and length of hospital stay were analyzed using the Pearson correlation analysis. In addition, we performed a multivariate linear regression analysis that included variables with a *P *value of less than 0.05 in the univariate analysis, except baseline spirometry. In addition, multivariate linear regression analysis was conducted using a backward elimination procedure. In the multivariate analysis, *B* was the regression coefficient, and a negative sign of the regression coefficient meant that the variables were negatively associated.

Third, we used receiver operating characteristic (ROC) curve analysis to predict the sensitivity and specificity of D_LCO,_ FEV_1_ and D_LCO_ + FEV_1_ as prognostic markers in severe AECOPD. When analyzing the ROC curve, D_LCO,_ FEV_1_ and D_LCO_ + FEV_1_ were analyzed as continuous variables. A *P *value of less than 0.05 was considered statistically significant.

## Results

### Characteristics of studied subjects

Among the 342 events, the D_LCO_ normal group comprised 227 events (the D_LCO_ value was more than 50% of the predicted value), and 115 in the D_LCO_ impaired group. In the FEV_1_ normal group (the FEV_1_ value was more than 50% of the predicted value), there was 173 events, and the FEV_1_ impaired group had 169 events. The average age was 71.5 ± 9.2 years. A total of 238 (69.6%) events were male and 104 (30.4%) were female. Sixty-three (18.4%) events were current smokers and the average pack/year history was 41.3 ± 17.1 years. A total of 225 (65.38) events were using inhalers, and 165 (48.2%) were taking respiratory-related oral medications. Averaged FEV_1_ was 1.3 ± 0.5 L (54.0 ± 19.3%) and D_LCO_ was 10.6 ± 4.8 L (59.3 ± 21.4%). (Table 1) In both groups, the average length of hospital stay was 10.0 ± 5.1 days. The mortality rate was 11 (3.2%), the experience of ventilator care was 29 (8.5%), and the experience of ICU care was 39 (11.4%).

**Table 1:** Baseline characteristics of patients with AECOPD

	D_LCO_ normal group (D_LCO_ > 50, n = 227)	D_LCO_ Impaired group (D_LCO_ ≤ 50, n = 115)	*P* value	FEV_1_ normal group (FEV_1_ > 50, n = 173)	FEV_1_ impaired group (FEV_1_ ≤ 50, n = 169)	*P *value	Total (n = 342)
Age (years)^†^	71.1 ± 9.5	72.4 ± 8.6	0.223	72.7 ± 9.8	70.4 ± 8.5	0.023	71.5 ± 9.2
*Sex, no. of exacerbations*							
Male^‡^	144 (63.4%)	94 (81.7%)	0.001	105 (60.7%)	133 (78.7%)	< 0.001	238 (69.6%)
Female^‡^	83 (36.6%)	21 (18.3%)		68 (39.3%)	36 (21.3%)		104 (30.4%)
*Smoking history, no. of exacerbations*							
Current smoker^‡^	42 (18.5%)	21 (18.3%)	0.957	32 (18.5%)	31 (18.3%)	0.971	63 (18.4%)
Ex-smoker^‡^	185 (81.5%)	94 (81.7%)		141 (81.5)	138 (81.7%)		279 (81.6%)
Pack-year history^†^	41.1 ± 16.8	41.8 ± 17.9	0.446	40.9 ± 16.5	41.7 ± 17.8	0.987	
*Comorbidities, no. of exacerbations*							
Hypertension^‡^	111 (48.9%)	53 (46.1%)	0.623	85 (49.1%)	79 (46.7%)	0.659	164 (48.0%)
Diabetes^‡^	54 (23.8%)	25 (21.7%)	0.671	43 (24.9%)	36 (21.3%)	0.436	49 (23.1%)
Previous TB history^‡^	58 (25.6%)	43 (37.4%)	0.023	35 (20.2%)	66 (39.1%)	< 0.001	101 (29.5%)
Coronary artery disease^‡^	37 (16.3%)	17 (14.8%)	0.716	32 (18.5%)	22 (13.0%)	0.165	54 (15.8%)
Cerebrovascular accident^‡^	6 (2.6%)	9 (7.8%)	0.027	5 (2.9%)	10 (5.9%)	0.172	15 (4.4%)
*Inhaler use before admission*							
LABAs^‡^	2 (0.9%)	1 (0.9%)	0.015	2 (1.2%)	1 (0.6%)	< 0.001	3 (0.9%)
LAMAs^‡^	24 (10.6%)	14 (12.2%)		27 (15.6%)	11 (6.5%)		38 (11.1%)
LABAs + LAMAs^‡^	36 (15.9%)	16 (13.9%)		24 (13.9%)	28 (16.6%)		52 (15.2%)
ICS/LABAs^‡^	25 (11.0%)	7 (6.1%)		21 (12.1%)	11 (6.5%)		32 (9.4%)
Triple therapy^‡^	53 (23.3%)	47 (40.9%)		32 (18.5%)	68 (40.2%)		100 (29.2%)
None^‡^	87 (38.3%)	30 (26.1%)		67 (38.7%)	50 (29.6%)		117 (34.2%)
*Oral medication before admission*							
Oral β2 adrenoreceptor agonist^‡^	8 (3.5%)	19 (16.5%)	< 0.001	9 (5.2%)	18 (10.7%)	0.062	27 (7.9%)
Roflumilast^‡^	7 (3.1%)	10 (8.7%)	0.024	1 (0.6%)	16 (9.5%)	< 0.001	17 (5.0%)
Mucolytic agent^‡^	92 (40.5%)	65 (56.5%)	0.005	68 (43.3%)	89 (52.7%)	0.013	157 (45.9%)
Oral steroids^‡^	6 (2.6%)	2 (1.7%)	0.722	2 (1.2%)	6 (3.6%)	0.170	8 (2.3%)
Oral antibiotics^‡^	7 (3.1%)	4 (3.5%)	1.000	3 (1.7%)	8 (4.7%)	0.116	11 (3.2%)
*Baseline spirometry*							
FEV_1_ (liters)^†^	1.5 ± 0.5	1.1 ± 0.4	< 0.001	1.6 ± 0.5	1.0 ± 0.3	< 0.001	1.3 ± 0.5
FEV_1_ (% of predicted value)^†^	59.9 ± 18.1	42.1 ± 16.0	< 0.001	69.5 ± 13.6	38.0 ± 8.1	< 0.001	54.0 ± 19.3
D_LCO_ (liters)^†^	12.5 ± 5.0	6.6 ± 2.2	< 0.001	11.9 ± 5.3	8.9 ± 4.1	< 0.001	10.6 ± 5.1
D_LCO_ (% of predicted value)^†^	73.5 ± 16.4	38.7 ± 8.8	< 0.001	71.4 ± 20.4	52.0 ± 18.7	< 0.001	61.8 ± 21.8

*AECOPD* acute exacerbations of chronic obstructive pulmonary disease, *LABAs* long acting B agonist bronchodilator, *LAMAs* long acting antimuscarinic agent bronchodilator, *ICS* inhaled corticosteroids, *FEV*_*1*_ forced expiratory volume in one second, *D*_*LCO*_ diffusing capacity of the lung for carbon monoxide

^†^Numbers are presented as mean ± standard deviation

^‡^Numbers are presented as n (%)

### Prognostic factor analysis classified using D_LCO_ and FEV_1_

**Fig. 2:**
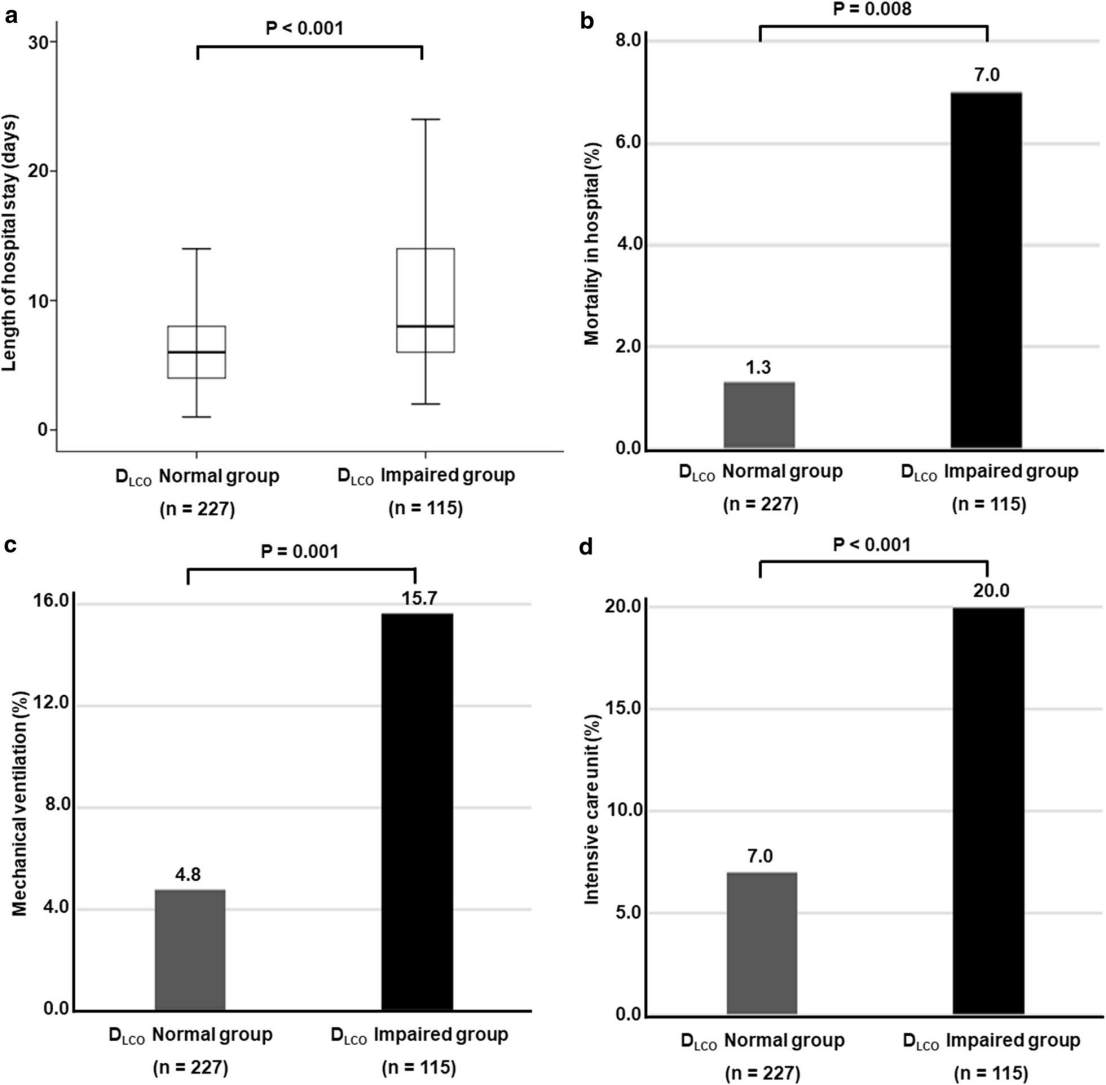
Prognosis analysis for severe AECOPD according to D_LCO_ classification. **a** Length of hospital stay (days), **b** mortality in hospital, **c** mechanical ventilation, and **d** intensive care unit. AECOPD, acute exacerbations of chronic obstructive pulmonary disease; D_LCO_, diffusing capacity of the lung for carbon monoxide

**Fig. 3:**
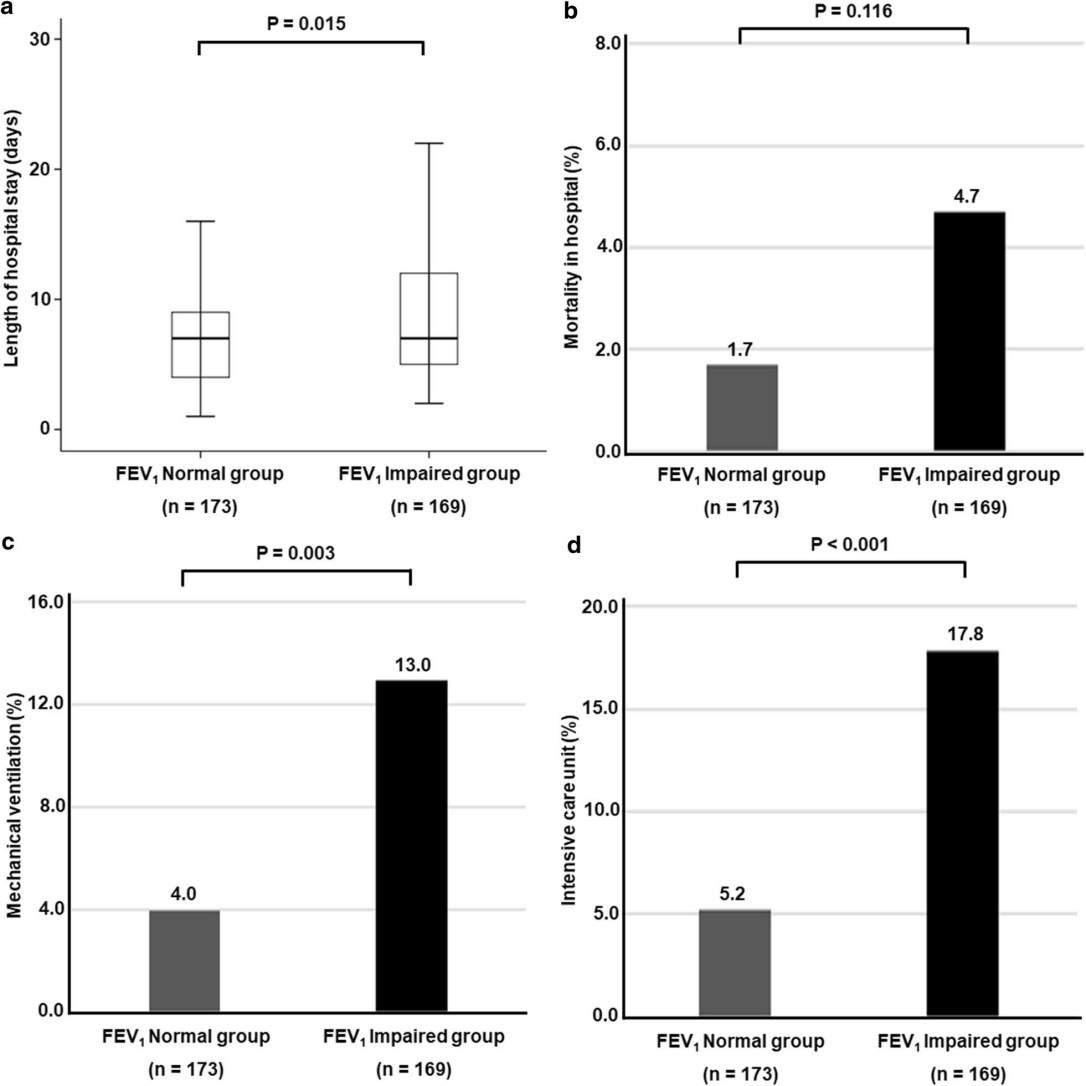
Prognosis analysis for severe AECOPD according to FEV_1_ classification. **a** Length of hospital stay (days), **b** mortality in hospital, **c** mechanical ventilation, and **d** intensive care unit. AECOPD, acute exacerbations of chronic obstructive pulmonary disease; FEV1, forced expiratory volume in one second

When classified through D_LCO_, the D_LCO_ impaired group showed a poor prognosis in all four factors by univariate analysis (Fig. 2). When classified through FEV_1_, the FEV_1_ impaired group showed a poor prognosis in three factors by univariate analysis (Fig. 3). However, there was no statistically significant mortality rate when classified as FEV_1_ (*P *value = 0.116) (Fig. 3B).

In multivariate analyses, D_LCO_ was associated with mortality (odds ratio = 4.408; 95% CI 1.070–18.167; *P* = 0.040) and need for mechanical ventilation (odds ratio = 2.855; 95% CI 1.216–6.704; *P* = 0.016) and ICU care (odds ratios = 2.685; 95% CI 1.290–5.590; *P* = 0.008). In severe AECOPD, D_LCO_ has been shown to predict mortality rate, ventilator, and ICU possibilities. When classified as FEV_1_, the experience of mechanical ventilation and ICU showed statistical significance. However, there was no significant difference in mortality rate (*P* = 0.075) (Table 2).

**Table 2:** Prognosis analysis for severe AECOPD

Parameter	Univariate analysis			Multivariate analysis		
	D_LCO_ normal group (D_LCO_ > 50, n = 227)	D_LCO_ impaired group (D_LCO_ ≤ 50, n = 115)	*P* value	Odds ratio	95% CI	*P* value
Mortality in hospital^**‡**^	3 (1.3%)	8 (7.0%)	0.008	4.408	1.070–18.167	0.040
Mechanical ventilation^**‡**^	11 (4.8%)	19 (15.7%)	0.001	2.855	1.216–6.704	0.016
Intensive care unit^**‡**^	16 (7.0%)	23 (20.0%)	< 0.001	2.685	1.290–5.590	0.008

	FEV_1_ normal group (FEV_1_ > 50, n = 173)	FEV_1_ impaired group (FEV_1_ ≤ 50, n = 169)				
Mortality in hospital^**‡**^	3 (1.7%)	8 (4.7%)	0.116	4.633	0.858–25.036	0.075
Mechanical ventilation^**‡**^	7 (4.0%)	22 (13.0%)	0.003	3.518	1.335–9.270	0.011
Intensive care unit^**‡**^	9 (5.2%)	30 (17.8%)	< 0.001	4.527	1.886–10.869	0.001

Multivariate analysis was conducted for variables with a *P *value of less than 0.05 in the univariate analysis, except for baseline spirometry

*AECOPD* acute exacerbations of chronic obstructive pulmonary disease, *FEV*_*1*_ forced expiratory volume in one second, *D*_*LCO*_ diffusing capacity of the lung for carbon monoxide

^†^Numbers are presented as mean ± standard deviation

^‡^Numbers are presented as n (%)

### Correlation analysis between spirometer factors and length of hospital stay

The length of hospital stay of the D_LCO_ normal group was 7.3 ± 5.0 days and the D_LCO_ impaired group was 12.4 ± 13.2 days. The length of hospital stay of the FEV_1_ normal group was 7.7 ± 5.4 days and the FEV_1_ impaired group was 10.4 ± 11.4 days. In the Pearson correlation analysis, both D_LCO_ and FEV_1_ showed a negative correlation. In multivariate linear regression analyses, D_LCO_ (*B* = − 0.542 ± 0.121, *P* < 0.001) and FEV_1_ (*B* = − 0.106 ± 0.106, *P* = 0.006) were negatively associated with length of hospital stay. Additionally, the regression coefficient was more pronounced in the D_LCO_ analysis (Table 3).

**Table 3:** Correlation analysis of length of hospital stay

Parameter	Univariate (Pearson correlation analysis)		Multivariate (multivariate linear regression analysis)		
	Correlation coefficient	*P* value	*B*	Standard deviation	*P* value
D_LCO_	− 0.272	< 0.001	− 0.542	0.121	< 0.001
FEV_1_	− 0.176	0.001	− 0.293	0.106	0.006

Multivariate analysis was conducted for variables with a *P *value of less than 0.05 in the univariate analysis, except for baseline spirometry. *B* is the regression coefficient, and the negative sign of the regression coefficient means that the variables are negatively associated

*FEV*_*1*_ forced expiratory volume in one second, *D*_*LCO*_ diffusing capacity of the lung for carbon monoxide

### ROC curve analysis of D_LCO_ and FEV_1_

**Fig. 4:**
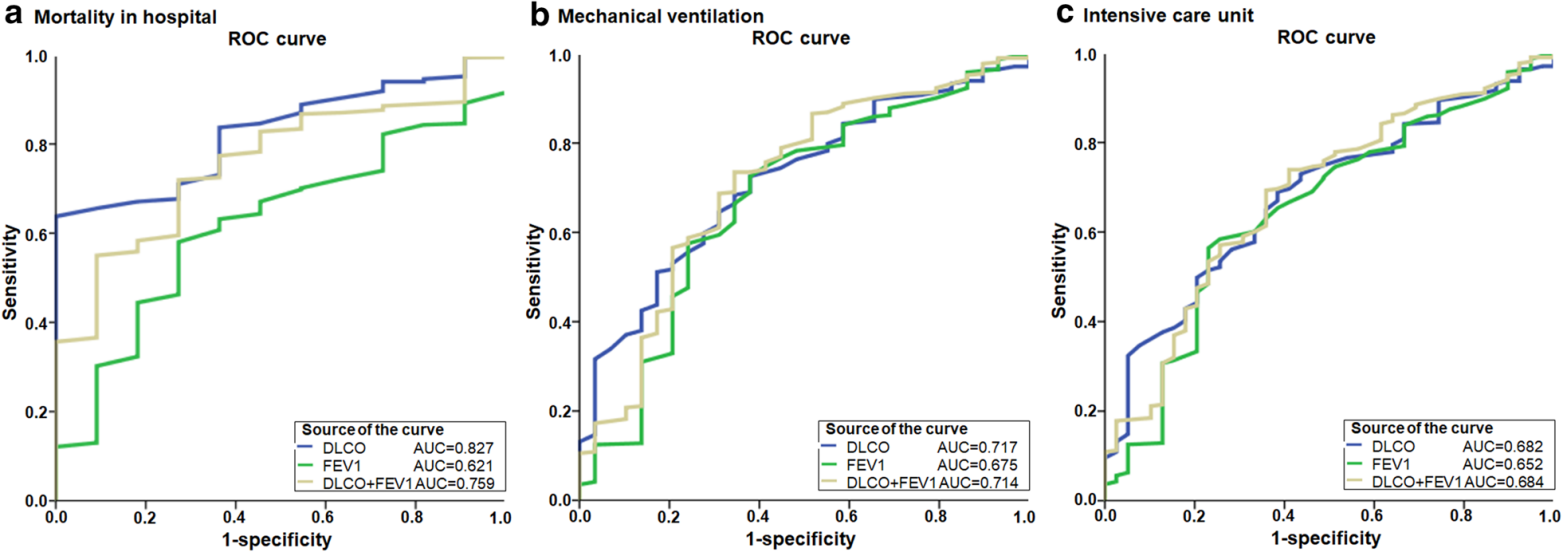
ROC curve of D_LCO_, FEV_1_, and D_LCO_ + FEV_1_. **a** Mortality in hospital, **b** mechanical ventilation, and **c** intensive care unit. ROC, receiver operating characteristics; FEV1, forced expiratory volume in one second; D_LCO_, diffusing capacity of the lung for carbon monoxide

When analyzing the sensitivity and specificity using the ROC curve, D_LCO_ showed better predictive ability than FEV_1_ (Table 4). When analyzing three prognostic factors (mortality in hospital, mechanical ventilation, and ICU care) through ROC curve analysis, area under the curve (AUC) was greater than 0.68 in all cases of D_LCO_ (Fig. 4). In contrast, the AUCs of FEV_1_ were below 0.68 in all three prognostic factors. In addition, the sensitivity and specificity of D_LCO_ were more than 64.1%, which was generally higher than FEV_1_. D_LCO_ + FEV_1_ showed similar values to D_LCO_.

**Table 4:** ROC curve analysis of D_LCO_, FEV_1_, and D_LCO_ + FEV_1_

Parameter	Prognostic factor	Optimal cut-off	Sensitivity	Specificity	AUC	95% confidence interval	*P* value
Mortality in hospital	D_LCO_	48.5	71.0	72.7	0.827	0.749–0.905	< 0.001
	FEV_1_	45.5	63.1	63.6	0.621	0.481–0.760	0.173
	D_LCO_ + FEV_1_	47.25	72.7	71.9	0.759	0.649–0.870	0.003
Mechanical ventilation	D_LCO_	51.5	68.4	65.5	0.717	0.629–0.804	< 0.001
	FEV_1_	44.5	66.5	65.5	0.675	0.566–0.784	0.002
	D_LCO_ + FEV_1_	50.25	69.0	68.7	0.714	0.612–0.816	< 0.001
Intensive care unit	D_LCO_	53.5	65.0	64.1	0.682	0.602–0.762	< 0.001
	FEV_1_	46.5	63.0	64.1	0.652	0.560–0.743	0.002
	D_LCO_ + FEV_1_	50.25	64.1	69.3	0.684	0.597–0.771	< 0.001

*ROC* receiver operating characteristics, *AUC* area under the curve, *FEV*_*1*_ forced expiratory volume in one second, *D*_*LCO*_ diffusing capacity of the lung for carbon monoxide

## Discussion

This is the study to compare FEV_1_ and D_LCO_ as prognostic markers in severe patients with AECOPD in Korea. In our study, the factors of prognosis were defined as the length of hospital stay, mortality rate in the hospital, experience of ventilation, and experience of ICU care. Classification by D_LCO_ showed significant differences in all prognostic factors. However, classification by FEV_1_ did not show a statistically significant mortality rate. The number of deaths was small, so caution is needed in the interpretation about death (the 95% confidence interval of the odds ratio was large and the *P *value was marginal). In the correlation analysis, both D_LCO_ and FEV_1_ showed a negative correlation with the length of hospital stay. The correlation coefficient was more pronounced in the D_LCO_ classification. In addition, when analyzing the ROC curve, D_LCO_ showed better predictive ability than FEV_1_. Of course, some odds ratio values were better when classified as FEV_1_ in our study. However, D_LCO_ was better in various analysis methods (correlation analysis, ROC curve analysis), which was likely to be as good as or better than FEV_1_.

The PFT has various parameters. In general, we used FEV_1_ to grade COPD and select the inhaler. In addition to FEV_1_, D_LCO_ is an important prognostic factor. In a study of smokers who did not show an obstruction pattern in PFT, a low D_LCO_ group showed quickly decreased pulmonary function and COPD progression [12]. Studies have shown that D_LCO_ is a more accurate prognostic factor than FEV_1_ when assessing postoperative risk [13, 14]. In addition, D_LCO_ is known to accurately represent the actual emphysema level and performance status [15, 16]. These results suggest that D_LCO_ can be a good predictor of early pulmonary dysfunction and prognosis.

If we know the prognosis of the patient early, we can focus on high-risk patients and improve the prognosis. The prognostic factors that can be used in the clinic are laboratory findings, scoring systems such as CAT or mMRC, and baseline spirometry [17, 18]. In some studies, high-C-reactive protein, eosinopenia, and thrombocytopenia are associated with poor outcomes in AECOPD [19–21]. Although various scoring systems—such as St. George's Respiratory Questionnaire, mMRC, and CAT, are useful—patients with severe symptoms may not be graded or might have similar scores, making them difficult to use. Instead, we focused on baseline spirometry and confirmed that D_LCO_ is more accurate in evaluating the prognosis of hospitalized patients than FEV_1_. If a grading system that considers both D_LCO_ and FEV_1_ is developed, the prognosis can be predicted more accurately.

Our study was limited because it was a retrospective single-center study. We were unable to analyze including important prognostic factors such as frequent exacerbations, obstructive sleep apnea, and body mass index. As this study is a retrospective study, data on these factors were not available or inaccurate. To compensate for this, we carefully analyzed the charts by two experienced pulmonologists. Also, we included as many factors as possible in baseline characteristics and multivariate analysis. In addition, the treatment received during the hospitalization period and the prognosis after discharge were not evaluated. Large prospective clinical studies that include information on treatment during hospitalization and post discharge may be required.

## Conclusion

D_LCO_ was likely to be as good as or better as a prognostic marker than FEV_1_ in severe AECOPD. Accurate classification using D_LCO_ may help to treat severe ACEOPD patients.

## Data Availability

The datasets used and/or analysed during the current study available from the corresponding author on reasonable request.

## Abbreviations

COPD: Chronic obstructive pulmonary disease
AECOPD: Acute exacerbation of chronic obstructive pulmonary disease
D_LCO_: Diffusing capacity of the lung for carbon monoxide
FEV_1_: Forced expiratory volume in one second
